# Nodakenin Induces ROS-Dependent Apoptotic Cell Death and ER Stress in Radioresistant Breast Cancer

**DOI:** 10.3390/antiox12020492

**Published:** 2023-02-15

**Authors:** Tae Woo Kim

**Affiliations:** Department of Biopharmaceutical Engineering, Dongguk University-WISE, 123 Dongdae-ro, Gyeongju 38066, Gyeongbuk, Republic of Korea; tae1410@naver.com

**Keywords:** nodakenin, Nox4, ER stress, apoptosis, ROS

## Abstract

*Angelica gigas* exerts powerful anti-tumor and anti-cancer effects in various cancer cell types. However, there have been few studies regarding the anti-cancer effect of nodakenin, a bioactive compound of *Angelica gigas*, in vivo and in vitro on breast cancers. I found that nodakenin, in a concentration-dependent manner, inhibits breast cancer cell viability and decreases the tumor volume in mice. Additionally, nodakenin induces caspase-3-dependent apoptosis in breast cancer cells; however, the combination of Z-VAD-FMK and nodakenin suppresses the caspase-3-dependent apoptotic cell death. Furthermore, nodakenin mediates apoptotic cell death via the PERK-mediated signaling pathway and calcium (Ca^2+^) release, and nodakenin combined with thapsigargin induces synergistic cell death by inhibiting sarco/endoplasmic reticulum (ER) Ca^2+^-ATPase. However, knockdown of PERK or CHOP inhibits Ca^2+^ generation and caspase-dependent apoptosis in nodakenin-treated breast cancer cells. Nodakenin induces ROS and Ca^2+^ generation, ER stress, and apoptotic cell death; however, the knockdown of Nox4 inhibits ROS generation and ER stress- and caspase-dependent apoptotic cell death. In addition, nodakenin combined with radiation overcomes radioresistance in radioresistant breast cancer cells by suppressing epithelial–mesenchymal transition phenotypes, including the decrease in E-cadherin and the increase in N-cadherin and vimentin. Therefore, these findings indicate that nodakenin may be a novel therapeutic strategy for breast cancers.

## 1. Introduction

Among females, breast cancer ranked the highest for mortality and incidence worldwide in 2020 [1]. Triple-negative breast cancer (TNBC) accounts for approximately 15–20% of all breast cancer diagnoses. TNBC is referred to as triple negative since it lacks the progesterone receptor, estrogen receptor, and human epidermal growth factor receptor 2 [2]. TNBC is the most lethal subtype of breast cancer and includes aggressive behavior and increased relapse, relapse, recurrence, and therapy failure rates [3]. Chemotherapy is the primary therapeutic strategy for TNBC patients; however, TNBC frequently develops resistance to the therapy [4]. To overcome this significant obstacle in TNBC treatments, a novel anti-cancer drug needs to be successfully developed for TNBC and breast cancers.

The endoplasmic reticulum (ER) is involved in the synthesis of cellular lipids, protein and lipid folding, protein maturation, free calcium (Ca^2+^) storage, reactive oxygen species (ROS) release, and the synthesis of secretory and membrane proteins [5]. The unfolded protein response (UPR) evokes intercellular communication from the ER to the cytosol and nucleus and regulates cellular homeostasis and pathogenesis in various diseases [6]. An imbalance and insufficient lipid and protein folding induce the ER stress response by accumulating misfolded proteins. Unfolded and misfolded proteins mediate UPR signaling by activating UPR sensors that reside on the ER membrane, activating transcription factor 6 (ATF6), PKR-like ER kinase (PERK), and inositol requiring enzyme 1α (IRE1α) [7,8,9]. Under diverse tumor environments, including hypoxia, nutrient deprivation, drug-induced toxicity, and radiation, accumulated unfolded and misfolded proteins contribute to the ER stress signaling pathway by releasing ROS [10]. The ER stress response by ROS production determines cell fate in many diseases such as diabetes and cancer, and excessive and continuous ER stress induces apoptotic cell death to eliminate defective cells [11]. Previous studies have indicated that intracellular Ca^2+^ release can be regulated by ROS production [12]. ROS release derived from NADPH oxidases has been shown to be involved in Ca^2+^ release [13]. The knockdown of NADPH oxidases (Nox) 4 expression inhibited ROS and Ca^2+^ release and induced apoptotic cell death and the ER stress response in anti-cancer drug-treated cancer cells [14]. Therefore, the crosstalk between intracellular ROS and Ca^2+^ regulates apoptotic cell death through cellular ER stress and UPR activation and may be a target for an effective tumor therapeutic strategy [15].

Although transmembrane protein UPR sensors bind to the major ER chaperone GRP78/Bip in the ER, the ER stress response inhibits this binding and activates signaling pathways through the phosphorylation of PERK, ATF6, and IRE1α to restore homeostasis [16]. The phosphorylation of the cytosolic sides of PERK and its downstream cascade (PERK/ATF4/CHOP) has been shown to induce cell death to restore cell homeostasis [17]. Upon the phosphorylation of PERK, PERK phosphorylates eukaryotic translation initiation factor 2α (eIF2α) in the cytosol, and this phosphorylation activates the transcription factor ATF4 to induce protein and amino acid synthesis and secretion and initiate the antioxidant stress response in the nucleus [18,19]. Upon translocation from the cytosol to the nucleus, ATF4 binds to the C/EBP-homologous protein (CHOP) promoter, and CHOP induces cell death [20]. Additionally, CHOP upregulates endoplasmic reticulum oxidoreduction 1 (ERO1α), creating a hyper oxidizing environment in the ER and leading to cell death in vitro and in vivo [21].

The use of traditional and natural medicine is frequently used for the treatment of various diseases. Recent research has used traditional and natural medicines as novel sources for treating malignancies [22,23]. *Angelica gigas Nakai* ethanol extracts have anti-cancer, anti- angiogenic, and anti-metastatic effects on various cancers and cancer models, including stomach, breast, non-small cell lung, prostate, and ovarian cancers [24,25,26,27]. The bioactive compounds extracted from *Angelica gigas* include decursin, umbelliferone, decursinol angelate, nodakenin, marmesin, peucedanone, and demethylsuberosin [28]. The major bioactive compounds of *Angelica gigas* extracts are decursin, decursinolangelate, and nodakenin [29]. Many studies have reported that decursin and decursinolangelate exert powerful anti-cancer effects through the activation of protein kinase C (PKC) in various human cancer cells [30]. Nodakenin is a coumarin compound and phytochemical isolated from the root of *Angelica gigas* and has been used to treat asthma and chronic bronchitis [31,32]. Previous studies have indicated that nodakenin suppresses inflammatory responses by inhibiting tumor necrosis factor receptor-6 (TRAF-6) and nuclear factor-κB (NF-κB) signaling in lipopolysaccharide-treated macrophages and inhibits allergic inflammation via the induction of caspase-1 and NF-κB cellular signaling pathways [33,34].

Therefore, I investigated if nodakenin exerts anti-cancer effects on breast cancer in vivo and in vitro. Furthermore, I studied whether nodakenin induces apoptosis and cell death via ROS generation in radioresistant breast cancer cells and identified nodakenin-mediated molecular mechanisms.

## 2. Materials and Methods

### 2.1. Reagents

Drugs were purchased as follows: nodakenin (Sigma Aldrich, St. Louis, MO, USA), Z-VAD-FMK (50 μM; Sigma Aldrich, St. Louis, MO, USA), diphenyleneiodonium (DPI, 1 μM; Sigma Aldrich, St. Louis, MO, USA), N-acetylcysteine (NAC, 100 μM; Sigma Aldrich, St. Louis, MO, USA), and thapsigargin (TG; Millipore, Burlington, MA, USA).

### 2.2. Cell Culture

The human normal breast cell line, MCF10A, was purchased from the American Type Culture Collection (ATCC, Manassas, VA, USA). These cells were incubated and cultured in Dulbecco’s modified Eagle medium nutrient mixture F-12 (DMEM/F12, Gibco, Waltham, MA, USA) supplemented with 10% fetal bovine serum (FBS, Welgene, Gyeongsan, Republic of Korea), epidermal growth factor (EGF; 20 ng/mL, Invitrogen, Waltham, MA, USA), hydrocortisone (0.5 μg/mL, Sigma), insulin (0.01 mg/mL, Sigma) 100 units/mL penicillin (Gibco), and 100 mg/mL streptomycin (Gibco). Human breast cancer cell lines (MCF7, SK-BR-3, T47D, HCC1419, HT20, and MDAMB231) were purchased from the Korean Cell Line Bank (Cancer Research Center, Seoul National University, Seoul, Republic of Korea). These cells were cultured in DMEM (Gibco, Waltham, MA, USA) supplemented with 10% FBS (Welgene, Gyeongsan, Republic of Korea), 100 units/mL penicillin (Gibco), and 100 mg/mL streptomycin (Gibco). The cells were incubated in a humidified incubator with 5% CO_2_ at 37 °C.

### 2.3. Cell Viability

All cell lines were plated into a 96-well plate with growth media at 1 × 10^4^ cells/well. After incubating for 24 h, the cells were treated with nodakenin for 24 h. A WST-1 assay (Sigma Aldrich, St. Louis, MO, USA) was performed and analyzed according to the manufacturer’s protocol.

### 2.4. LDH Assay

All cell lines were plated into a 96-well plate with growth media at 1 × 10^4^ cells/well and allowed to grow for 24 h, the cells were treated with nodakenin for 24 h. An LDH cytotoxicity assay (Abcam, Milpitas, CA, USA) was performed and analyzed according to the manufacturer’s protocol.

### 2.5. Caspase-3 Activity Assays

MCF-7 and MDAMB231 cells were plated into a 6-well plate with growth media at 3 × 10^5^ cells/well for 24 h. After 24 h, the cells were treated with nodakenin for 24 h. A caspase-3 activity assay (Biovision colorimetric caspase-3 assay kit) was performed and analyzed according to the manufacturer’s protocol.

### 2.6. Intracellular Ca^2+^ Assays

MCF-7 and MDAMB231 cells were plated into a 96-well plate with growth media at 1 × 10^4^ cells/well. After 24 h, the cells were treated with nodakenin for 24 h. An intracellular calcium activity assay (Abcam, Ca^2+^ assay kit (Colorimetric)) was performed and analyzed as described in the supplier’s manual.

### 2.7. Intracellular ROS Assays

MCF-7 and MDA-MB-231 cells were plated into a 96-well plate with growth media at 1 × 10^4^ cells/well. After 24 h, the cells were treated with nodakenin for 24 h. Then, the cells were incubated with cell-permeant 2′7′-dichlorodihydrofluorescein diacetate (CM-H_2_DCFDA, Invitrogen) for 30 min at 37 °C, as described in the supplier’s protocol.

### 2.8. Establishment of Radioresistant MCF-7 and MDAMB231 Cells

MCF-7 and MDAMB231 cells were plated into 60 mm cell culture dishes at 3 × 10^5^ cells/dish. For radiation exposure, after 24 h, the cells were exposed to radiation at 4 Gy daily for 12 weeks. To generate radioresistant MCF-7 and MDAMB231 cells, the radiation exposure process was repeated until radioresistant cells (MCF-7R and MDAMB231R) were established.

### 2.9. Irradiation

MCF-7, MCF-7R, MDAMB231, and MDAMB231R cells were plated into 60 mm dishes with growth media at 3 × 10^5^ cells/dish and incubated at 37 °C with CO_2_ for 24 h. The cells were then exposed to radiation from ^137^Cs source irradiation (Atomic Energy of Canada, Ltd., Mississauga, ON, Canada). Established MCF-7R and MDAMB231R cells were exposed to a dose of 4 Gy for 90 days and then cultured in growth media containing 10% FBS.

### 2.10. Colony Formation Assay

MCF-7, MCF-7R, MDAMB231, and MDAMB231R cells were plated into 60 mm dishes with growth media at 3 × 10^5^ cells/dish. The cells were incubated for 10 days, and then the colonies were stained with 0.5% crystal violet (Sigma Aldrich, St. Louis, MO, USA). Crystal violet staining was performed and analyzed as described in the supplier’s manual.

### 2.11. Transfection

Small interfering RNAs (siRNAs) were purchased targeting PERK (Santacruz, Dallas, TX, USA), Nox4 (Santacruz), and CHOP (Bioneer, Daejeon, Korea). MCF-7 and MDAMB231 cells were plated into a 6-well plate at 3 × 10^5^ cells/well, and then the cells were transfected with siRNAs (30 nmol/mL) using Lipofectamine 2000 reagent (Invitrogen) according to the manufacturer’s protocol.

### 2.12. RNA and Protein Extraction

MCF-7 and MDAMB231 cells were plated into a 100 mm cell culture dish with growth media at 1 × 10^6^ cells/dish. The total RNA from the cells was extracted using Trizol reagent (Invitrogen) according to the supplier’s protocol. Protein from the cell lysates was collected using radioimmunoprecipitation assay (RIPA) buffer (Thermo Scientific, Waltham, MA, USA).

### 2.13. Real-Time qRT PCR and Western Blot Analyses

For the real-time qRT-PCR analysis, triplicate reactions were performed. The primer sequences used are as follows: GRP78 (5′-TCAGCCCACCGTAACAAT-3′ [sense] and 5′-CAAACTTCTCGGCGTCAT-3′ [antisense]) (NC_000009), ATF4 (5′-AAGCCTAGGTCTCTTAGATG-3′ [sense] and 5′-TTCCAGGTCATCTATACCCA-3′ [antisense)]) (NM_001675), CHOP (5′- ATGAGGACCTGCAAGAGGTCC-3′ [sense] and 5′-TCCTCCTCAGTCAGCCAAGC-3′ [antisense]) (NM_004083), E-cadherin (5′-GAACGCATTGCCACATACAC-3′ [sense] and 5′-GAATTCGGGCTTGTTGTCAT-3′ [antisense]) (NM_004360), N-cadherin (5′-GGCATACACCATG CCATCTT-3′ [sense] and 5′-GTGCATGAAGGACAGCCTCT-3′ [antisense]) (NM_001792), and vimentin (5′-GAGAACTTTGCCGTTGAAGC-3′ [sense] and 5′-GCTTCCTGTAGGTGGCAATC-3′ [antisense]) (NM_003380) on a LightCycler 96 System (Roche). RNA quantities were normalized with β-actin primer (5′-AAGGCCAAC CGCGAGAAGAT-3′ [sense] and 5′-TGATGACCTGGCCGTCAGG-3′ [antisense]), and the relative fold change for gene expression analysis was calculated using the 2^−ΔΔCt^ method. To identify protein expression using Western blot analysis, cells were lysed in RIPA buffer (Thermo Scientific). The membranes were blocked with 5% skim milk and then incubated with primary antibodies. The primary antibodies used were GRP78 (Santa Cruz), eIF2ᾳ (Santa Cruz), β-actin (Santa Cruz), p-eIF2ᾳ (Ser51) (Cell Signaling, Danvers, MA, USA), Nox4 (Proteintech, Rosamond, Illinois, USA). p-PERK(Thr980) (Cell Signaling), cleaved caspase-9 (Cell Signaling), PERK (Cell Signaling), CHOP (Cell Signaling), cleaved caspase-3 (Cell Signaling), and ATF4 (Cell Signaling). The membranes were then incubated with the HRP-conjugated secondary antibodies: m-IgGK BP-HRP-linked antibody (Santa Cruz) and anti-mouse anti rabbit IgG HRP-linked antibody (Santa Cruz). The membranes were visualized using a chemiluminescent HRP substrate (Millipore, Burlington, MA, USA).

### 2.14. Animal Experiments

Five-week-old female, athymic BALB/c nude mice (*nu*/*nu*) were purchased from OrientBio, Inc. (Daejeon, Republic of Korea), and housed in a pathogen-free room for one week with NIH-7 open formula. The mice were divided randomly into three groups. All animal studies were performed according to the guidelines of the National Institutes of Health and Kyung-Hee University Animal Care and Use Committee. For the xenograft mice experiment, 1 × 10^7^ MDAMB231 cells were mixed in PBS, and injected subcutaneously (sc) into the right dorsal flank of nude mice. When the tumor volume reached approximately 200 mm^3^, the mice were randomly grouped (*n* = 10 per group). Nodakenin (10 or 30 mg/kg) was then administered intraperitoneally (ip) twice weekly. Tumor volume was calculated using the following formula: (*L* × *W*^2^)/2 (mm^3^).

### 2.15. Statistical Analysis

Experimental data were confirmed by at least three independent experiments and all statistical analyses were performed using a student’s *t*-test; *p*-values less than 0.05 were considered to be statistically significant.

## 3. Results

### 3.1. Nodakenin Exerts an Anti-Tumor Effect in Breast Cancer Cell Lines

**Figure 1:**
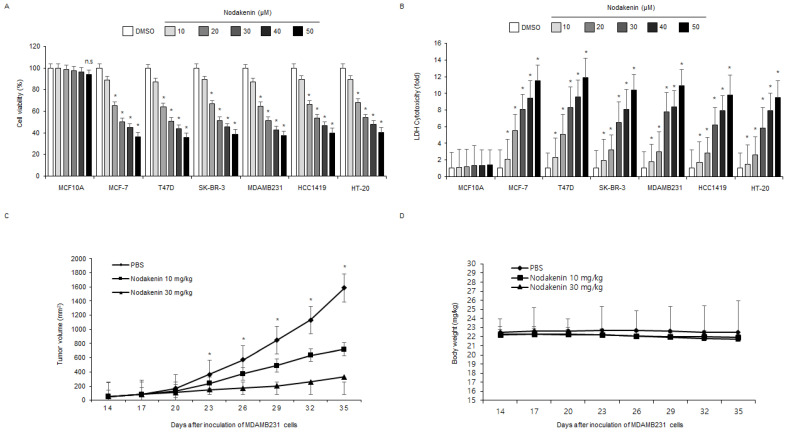
Anti-tumor effects of nodakenin in various breast cancer cell types and xenograft models. (**A**,**B**) Cell viability and LDH cytotoxicity were determined using the WST-1 assay and LDH cytotoxicity assay in a dose-dependent manner in nodakenin-induced normal breast and breast cancer cells (MCF10A, MCF-7, T47D, SK-BR-3, MDAMB231, HCC1419, and HT-20); * *p* < 0.05. n.s., not significant. (**C**,**D**) 1 × 10^7^ MDAMB231 cells were injected (sc) into the right dorsal flank of nude mice (*n* = 10/group). Nodakenin (10 or 30 mg/kg) was administered (ip) twice weekly. Body weights of the MDAMB231 tumor-xenograft mice were determined twice a week during the experiment.

**Figure 2:**
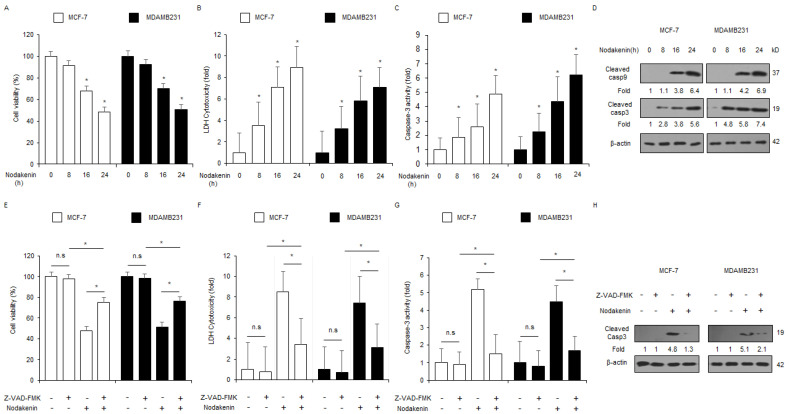
Nodakenin induces apoptosis and cell death in breast cancer cells. (**A**–**D**) After nodakenin was treated at various time points in breast cancer cells (0, 8, 16, and 24 h; 40 µM), biological experiments, such as LDH, WST-1, and caspase-3 activity assays were carried out. Western blot analyses were conducted for caspase-3 and -9 cleavage according to the indicated times in nodakenin-induced MCF-7 and MDAMB231 cells; * *p* < 0.05. β-actin was used as a protein-loading control. (**E**–**H**) MCF-7 and MDAMB231 cells were pre-treated with Z-VAD-FMK (50 μM) for four hours and were subsequently treated with nodakenin (40 µM, 24 h). Biological experiments, such as LDH, WST-1, and caspase-3 activity assays were carried out; * *p* < 0.05. n.s., not significant. Western blot analyses were performed with a cleaved caspase-3 antibody. β-actin was used as a loading control. Western blot analyses were confirmed in at least three independent experiments.

To investigate the anti-tumor effect of nodakenin in normal breast cells (MCF10A) and breast cancer cells (MCF-7, SK-BR-3, T47D, MDAMB231, HCC1419, and HT-20), I determined cell viability and cytotoxicity using WST-1 and LDH assays in a concentration-dependent manner (nodakenin; 10, 20, 30, 40, and 50 µM) (Figure 1A,B). To confirm the anti-tumor effects of nodakenin, a xenograft model using nude mice was established with TNBC MDAMB231 cells. Nodakenin injections at 10 mg/kg and 30 mg/kg showed smaller tumor volumes compared with the control (Figure 1C). In all groups, no significant effect on body weight was observed with nodakenin treatment (Figure 1D). To identify the inhibitory effect of nodakenin on tumor growth at various time points, breast cancer cell lines MCF-7 and MDAMB231 were treated with nodakenin for 0, 8, 16, and 24 h. I then assessed cell viability, cell cytotoxicity, and caspase-3 activity using WST-1, LDH, and caspase-3 activity assays, respectively. Nodakenin treatment inhibited cell viability and enhanced LDH release and caspase-3 activity in a time-dependent manner in MCF-7 and MDAMB231 cell lines (Figure 2A–C). Western blot analyses showed that nodakenin treatment induced caspase-3 and -9 cleavage (Figure 2D). To confirm whether nodakenin modulates apoptotic cell death by caspases in both cell lines, I performed an additional experiment using caspase inhibitor Z-VAD-FMK. Z-VAD-FMK alone caused no change in cell viability, LDH cytotoxicity, and caspase-3 activity; however, nodakenin alone inhibited cell viability and enhanced LDH cytotoxicity and caspase-3 activity. A combined treatment with nodakenin and Z-VAD-FMK dramatically suppressed the inhibition of cell viability and enhancement of LDH cytotoxicity and caspase-3 activity (Figure 2E–G). Moreover, Western blot analyses showed that Z-VAD-FMK and nodakenin combined blocked cleaved caspase-3 compared with nodakenin alone (Figure 2H). Our results suggest that nodakenin induces apoptosis and cell death in breast cancer cells.

### 3.2. Nodakenin Treatment Mediates Apoptosis through ER Stress in Breast Cancer

**Figure 3:**
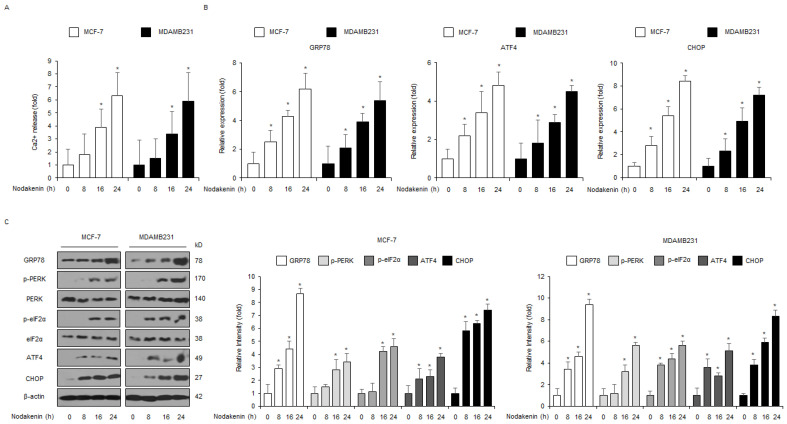
Nodakenin mediates the ER stress response through intracellular Ca^2+^ generation. (**A**) MCF-7 and MDAMB231 cells were treated with nodakenin at various time points (0, 8, 16, and 24 h; 40 µM) and then subjected to an intracellular Ca^2+^ assay. (**B**) MCF-7 and MDAMB231 cells were treated with nodakenin at various time points (0, 8, 16, and 24 h; 40 µM) and then mRNA expression levels of GRP78, ATF4, and CHOP were measured using real-time qRT-PCR. β-actin was used as a loading control. (**C**) MCF-7 and MDAMB231 cells were treated with nodakenin (0, 8, 16, and 24 h; 40 µM), and then the cellular signaling axis for the activation of ER stress was assessed by measuring the expression of ER stress-related proteins, such as GRP78, CHOP, ATF4, p-PERK, and p-eIF2α using Western blot analyses. β-actin was used as a protein-loading control. Western blot analyses were confirmed in at least three independent experiments. * *p* < 0.05.

Calcium (Ca^2+^) storage in the ER plays a central role in pro-survival and cell death by regulating various intracellular signaling pathways [35]. In addition, excessive ROS and intracellular Ca^2+^ release induces apoptosis in diverse cancers; thus, targeting ROS and Ca^2+^ is a potential anti-cancer therapeutic strategy [36,37]. Moreover, accumulating reports suggest that ROS and Ca^2+^ generation regulate cell death by inducing the ER stress signaling pathway [38]. Here, I showed that nodakenin mediated the release of intracellular Ca^2+^ in MCF-7 and MDAMB231 cells in a time-dependent manner (Figure 3A). To investigate the mRNA levels of ER stress-related markers, including GRP78, ATF4, and CHOP, I performed real-time qRT-PCR in nodakenin-treated MCF-7 and MDAMB231 cell lines. Nodakenin increased the mRNA levels of ATF4, GRP78, and CHOP at various time points to a greater extent than the control treatment (Figure 3B). To further assess the expression of ER stress-related proteins, such as GRP78, PERK, p-PERK, eIF2α, p-eIF2α, CHOP, and ATF4, in nodakenin-treated MCF-7 and MDAMB231 cells at various time points, i carried out Western blot analyses. Nodakenin time-dependently upregulated the protein expression of GRP78, p-PERK, p-eIF2α, ATF4, and CHOP to a greater extent than the control (Figure 3C).

### 3.3. Nodakenin, in Combination with Thapsigargin Mediates the Synergistic ER Stress Response in Breast Cancer

**Figure 4:**
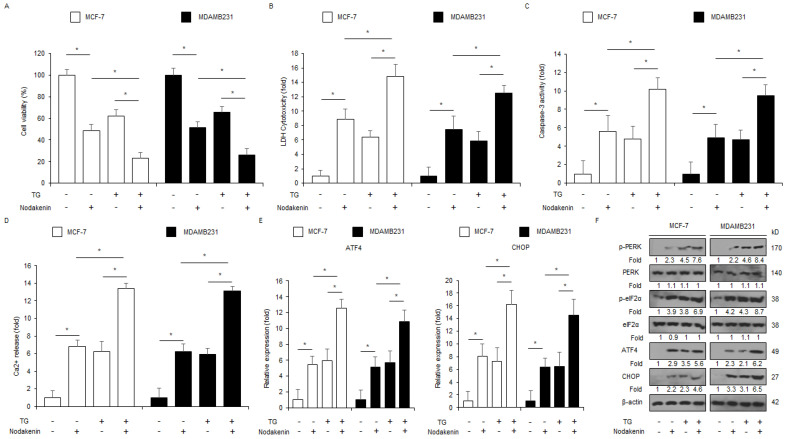
Nodakenin, in combination with thapsigargin, mediates a synergistic ER stress response through intracellular Ca^2+^ generation. (**A**–**D**) MCF-7 and MDAMB231 cells were co-treated with nodakenin (40 µM, 24 h) and thapsigargin (TG; 3 μM, 24 h), and then cell viability, LDH cytotoxicity, caspase-3 activity, and intracellular Ca^2+^ assays were carried out; * *p* < 0.05. (**E**,**F**) Real-time qRT-PCR of CHOP and ATF4 mRNA and Western blot analyses of p-PERK and p-eIF2α, CHOP, and ATF4 protein expression in nodakenin (40 µM, 24 h) and thapsigargin (TG; 3 μM, 24 h)-treated MCF-7 and MDAMB231 cell lines. β-actin was used as a loading control. Western blot analyses were confirmed in at least three independent experiments.

A prolonged ER stress response induced by natural products exerts an anti-cancer effect by triggering the apoptosis signaling pathway and is thus a powerful anti-cancer strategy [39]. To evaluate if ER stress was related to nodakenin-induced cell death, I used thapsigargin (TG, ER stress inducer) together with nodakenin to check cell viability, LDH cytotoxicity, caspase-3 activity, intracellular Ca^2+^ release, and PERK signaling pathway protein expression. As shown in Figure 4A–E, nodakenin effectively induced the synergistic inhibition of cell viability, release of LDH cytotoxicity and intracellular Ca^2+^, phosphorylation of PERK and eIF2α, and upregulation of CHOP and ATF4 in TG-induced MCF-7 and MDAMB231 cell lines.

### 3.4. Loss of PERK Blocks Nodakenin-Mediated Cell Death in Breast Cancer

**Figure 5:**
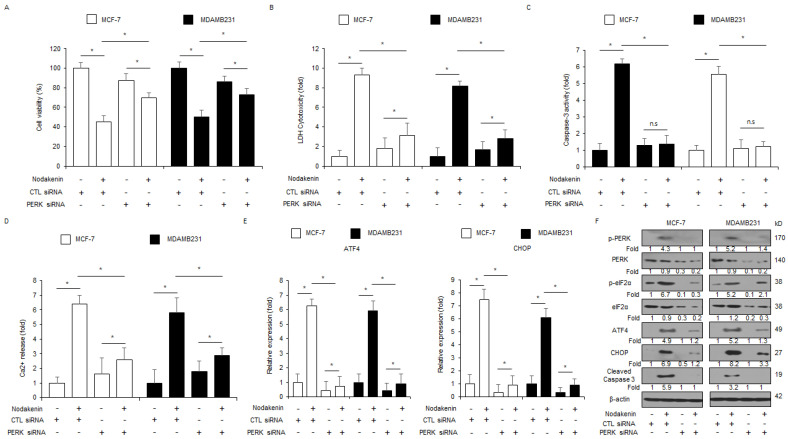
Inhibition of PERK suppresses nodakenin-induced apoptosis and cell death in breast cancer. (**A**–**D**) MCF-7 and MDAMB231 cells were transfected with PERK siRNA in the presence or absence of nodakenin (40 µM, 24 h) and WST-1, caspase-3 activity, LDH cytotoxicity, and intracellular Ca^2+^ assays were carried out; * *p* < 0.05. (**E**,**F**) Real-time qRT-PCR for CHOP and ATF4 mRNA expression levels and Western blot analysis of protein expression levels of the p-PERK, p-eIF2α, PERK, eIF2α, CHOP, ATF4, and Caspase-3 cleavage in nodakenin (40 µM, 24 h)-treated MCF-7 and MDAMB231 cells in the presence or absence of PERK siRNA (30 nM, 24 h). β-actin was used as the mRNA and protein-loading controls. Western blot analyses and real-time RT-PCR were confirmed in at least three independent experiments.

**Figure 6:**
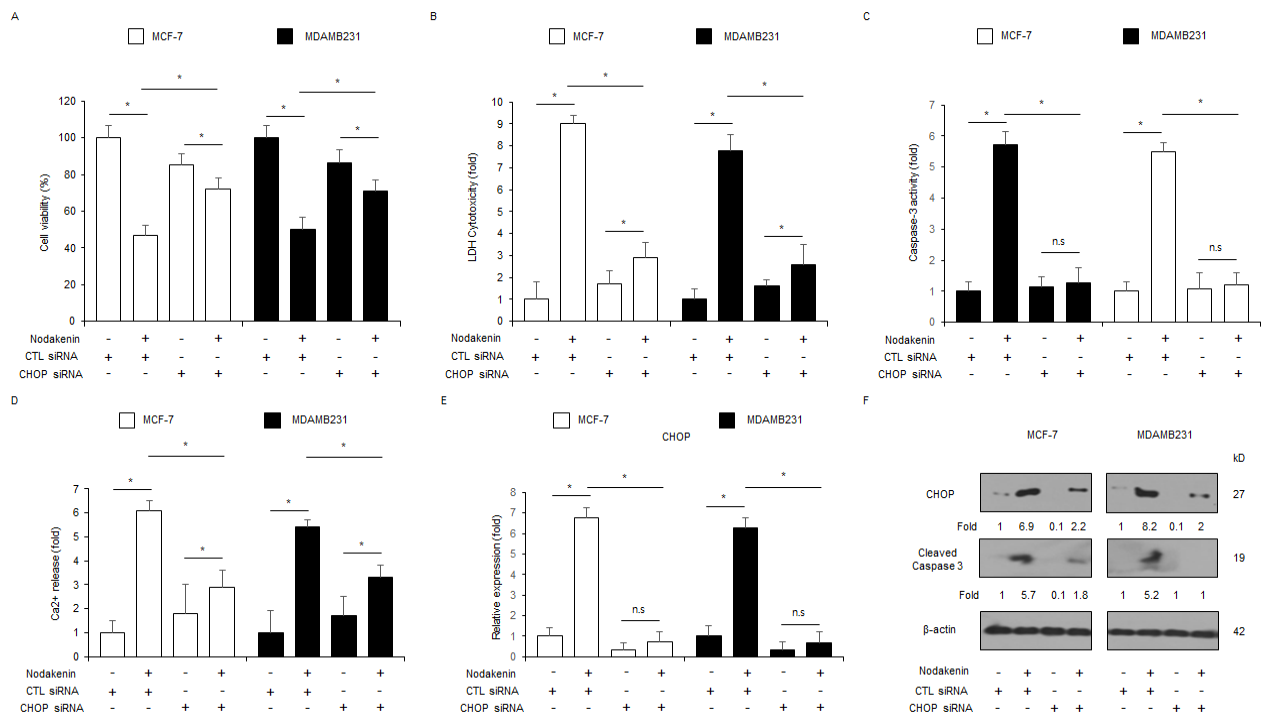
Inhibition of CHOP suppresses nodakenin-mediated apoptosis and cell death in breast cancer. (**A**–**D**) MCF-7 and MDAMB231 cells were transfected with CHOP siRNA with nodakenin (40 µM, 24 h) treatment, and LDH cytotoxicity, WST-1, caspase-3 activity, and Ca^2+^ assays were carried out; * *p* < 0.05. n.s., not significant. (**E**,**F**) Real-time qRT-PCR for CHOP expression levels and Western blot analyses for CHOP and caspase-3 cleavage levels in nodakenin (40 µM, 24 h)-treated MCF-7 and MDAMB231 cells with CHOP siRNA (30 nM, 24 h). β-actin was used as the mRNA and protein-loading controls. Western blot analyses and real-time RT-PCR were confirmed in at least three independent experiments.

I next determined the effect of PERK knockdown in nodakenin-treated breast cancer cell lines (MCF-7 and MDAMB231). The suppression of PERK using specific siRNAs blocked the inhibition of cell viability and the enhancement of LDH cytotoxicity, caspase-3 activity, Ca^2+^ production, the phosphorylation of PERK and eIF2α, and the upregulation of ATF4, CHOP, and cleaved caspase-3 in nodakenin-induced MCF-7 and MDAMB231 cell lines (Figure 5A–F). To further confirm if nodakenin induces cell death through the ER stress response, CHOP siRNAs were transfected into MCF-7 and MDAMB231 cell lines, which were then treated with nodakenin. Knockdown of CHOP suppressed the inhibition of cell viability, the enhancement of caspase-3 activity, LDH cytotoxicity, and Ca^2+^ production, and the upregulation of cleaved caspase-3 and CHOP compared to the control cells (Figure 6A–F). These results indicate that loss of ER stress markers, such as PERK and CHOP, suppresses apoptotic cell death through the ER stress cellular signaling axis in nodakenin-induced breast cancer cell lines.

### 3.5. Loss of Nox4 Blocks Nodakenin-Mediated ER Stress and Apoptotic Cell Death in Breast Cancer

**Figure 7:**
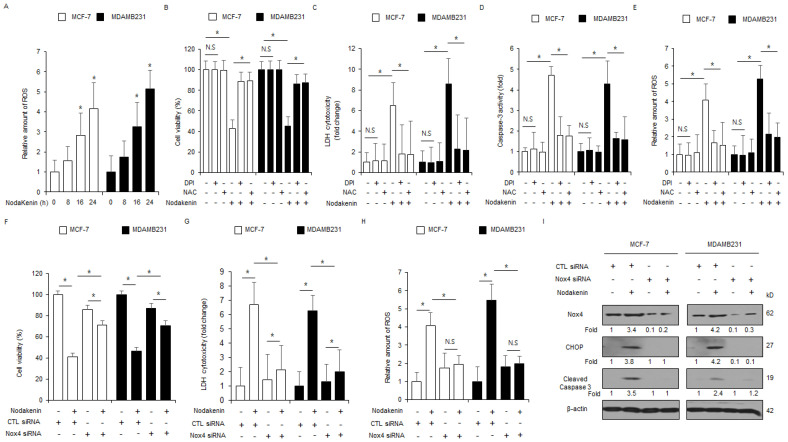
Inhibition of Nox4 suppresses nodakenin-mediated ROS production and ER stress in breast cancer. (**A**) ROS experiment by DCFDA dye performed with FACS analysis in nodakenin (40 µM, 24 h)-treated MCF-7 and MDAMB231 cells; * *p* < 0.05. (**B**–**E**) MCF-7 and MDAMB231 cells were treated with nodakenin (40 μΜ, 24 h), NAC (100 μM, 24 h), and DPI (1 μM, 24 h). Then LDH cytotoxicity, WST-1, ROS, and caspase-3 activity assays were performed; * *p* < 0.05. N.S., not significant. (**F**–**I**) MCF-7 and MDAMB231 cells were transfected with Nox4 siRNAs and treated with nodakenin (40 μM, 24 h). WST-1, LDH cytotoxicity, and intracellular ROS assays were performed along with Western blot analyses for CHOP, Nox4, and cleaved caspase-3; * *p* < 0.05. N.S., not significant. Western blot analyses were confirmed in at least three independent experiments.

To assess whether nodakenin treatment increases ROS production in breast cancer cells, a ROS assay was performed. Nodakenin enhanced intracellular ROS production in a time-dependent manner (Figure 7A). To verify whether NAC and DPI, Nox and ROS inhibitors, block nodakenin-mediated ROS production, ER stress, and apoptotic cell death in MCF-7 and MDAMB231 cells, i performed an intracellular ROS assay, WST-1 assay, and LDH assay. Nodakenin, in combination with DPI or NAC, suppressed the decrease in cell viability and enhanced LDH and ROS release to a greater extent than nodakenin treatment alone (Figure 7B–E). These results suggest that nodakenin treatment induces ER stress and apoptotic cell death by releasing ROS in breast cancer cells. To confirm if nodakenin regulates Nox4 expression and ROS generation, Nox4-specific siRNAs were transfected into MCF-7 and MDAMB231 cells, which were then treated with nodakenin. Nox4 knockdown led to a higher cell viability and lower LDH and ROS release in JI017-treated MCF-7 and MDAMB231 cells in comparison to the control (Figure 7F–H). Western blot analyses showed that Nox4 knockdown decreased Nox4, CHOP, and caspase-3 cleavage levels to a greater extent in nodakenin-treated MCF-7 and MDAMB231 cells than in the control cells (Figure 7I). These results indicate that Nox4 regulates apoptotic cell death and ER stress by upregulating ROS production in nodakenin-treated breast cancer cells.

### 3.6. Combined Treatment of Radiation and Nodakenin Overcomes Radioresistance via the Inhibition of the EMT Process in Radioresistant Breast Cancer Models

**Figure 8:**
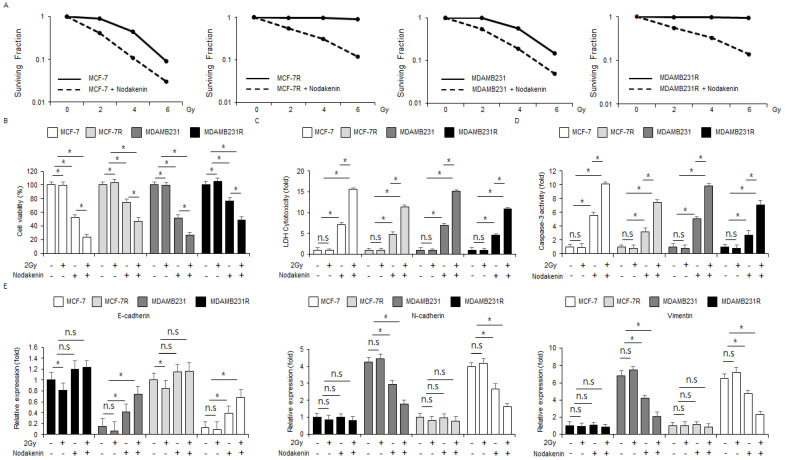
Radiation in combination with nodakenin overcomes resistance in radioresistant breast cancer models. (**A**) A colony formation assay was carried out at the indicated doses (0, 2, 4, and 6 Gy) of radiation in combination with nodakenin (40 µM), and then the survival fraction was calculated in MCF-7, MCF-7R, MDAMB231, and MDAMB231R; * *p* < 0.05. (**B**–**E**) MCF-7, MCF-7R, MDAMB231, and MDAMB231R cells were co-treated with nodakenin (40 µM, 24 h) and radiation (2 Gy, 24 h). LDH, WST-1, and caspase-3 activity assays were carried out along with a real-time qRT-PCR for the mRNA expression levels of E-cadherin, vimentin, and N-cadherin; * *p* < 0.05. n.s., not significant. β-actin was used as the mRNA loading control. Real-time RT-PCR was confirmed in at least three independent experiments.

To identify if 2 Gy combined with nodakenin overcomes radioresistance, radioresistant models (MCF-7R and MDAMB231R cells) were established by exposure to radiation in MCF-7 and MDAMB231 cell lines. I then performed the WST-1 assay, caspase-3 activity assay, colony formation assay, LDH assay, and real-time qRT-PCR. The findings showed that nodakenin (30 µM, 24 h) inhibited surviving fraction levels at the indicated conditions (2, 4, and 6 Gy) in MCF-7, MCF-7R, MDAMB231, and MDAMB231R cells when compared with the control cells (Figure 8A). Nodakenin inhibited cell viability and enhanced LDH release and caspase-3 activity in MCF-7 and MDAMB231 cells when compared with MCF-7R and MDAMB231R cells. Nodakenin with radiation mediated lesser cell viability and greater LDH cytotoxicity production and caspase-3 activity in MCF-7 and MDAMB231 cell lines when compared with MCF-7R and MDAMB231R models (Figure 8B–D). To determine if Nodakenin and radiation inhibited the EMT phenomenon in radioresistant breast cancer cell lines, I performed real-time qRT-PCR. In MCF-7 and MDAMB231 cells, nodakenin, radiation, and nodakenin with radiation showed no change in mRNA levels of EMT markers (E-cadherin, N-cadherin, and vimentin) (Figure 8E). However, the control or radiation alone showed downregulated E-cadherin and upregulated vimentin and N-cadherin in MCF-7R and MDAMB231R models. Nodakenin and nodakenin with radiation showed increased E-cadherin levels and decreased vimentin and N-cadherin compared to the control (Figure 8E).

## 4. Discussion

Accumulating studies reported that natural products may potentially function as powerful anti-cancer agents for cancer therapy [40,41]. High ROS and Ca^2+^ generation regulates the anti- or pro-oxidant defense system in cancer cells, and targeting oxidants could be a novel tumor therapeutic strategy [42,43]. I suggest that nodakenin is a promising anti-tumor reagent in radioresistant breast cancer models. In the present study, I investigated the detailed signaling pathways underlying apoptosis and cell death in nodakenin-treated breast cancer cells. Our results herein identified that nodakenin induced apoptotic cell death and the ER stress response by producing intracellular ROS and Ca^2+^ in nodakenin-treated MCF-7 and MDAMB231 cells. Nodakenin mediated apoptosis and cell death by inducing the PERK-dependent axis. Nox4 activation frequently induces ER stress and apoptotic cell death via ROS release [44,45]. Nox4 activation by nodakenin treatment induced ROS and Ca^2+^ release, ER stress, and apoptotic cell death in breast cancer cell lines; however, Nox4 knockdown blocked ER stress and apoptotic cell death via ROS and Ca^2+^ release in nodakenin-treated breast cancer cell lines. Moreover, nodakenin, in combination with radiation, overcame radioresistance by blocking the EMT process in radioresistant breast cancer models.

Traditional herbal medicines exert a powerful anti-cancer effect with fewer side effects [46]. Furthermore, synergistic effects induced by complex herbal extracts may suggest a novel anti-tumor therapeutic strategy in various chemo-resistant cancer cell lines [47]. The complex herbal medicine H3 + gemcitabine has anti-cancer effects, including G0/G1 phase cell cycle arrest, inhibition of migration, and cytochrome C release, in gemcitabine-resistant pancreatic cancer cells [48]. In our previous study, a herbal medicine complex, including SH003 and JI017, showed powerful anti-cancer effects in various cancer models. SH003 exerts apoptosis, ER stress, G9a-mediated epigenetic modification, and Bnip3-induced autophagic cell death via the release of ROS on hypoxia exposure in gastric cancer cells [25]. In addition, JI017 induces apoptosis and ER stress and overcomes paclitaxel resistance by increasing ROS and Nox4 expression in breast cancer cells [23]. JI017 also induces potential anti-cancer effects through the increase in ER stress and the release of intracellular ROS in radioresistant ovarian cancer models and mouse models [27]. Both SH003 and JI017 contain *Angelica gigas*. Among Angelica gigas, nodakenin, decursin, and decursinolangelate are known as active compounds [28]. Although there are many reports of the anti-cancer effects of decursin and decursinolangelate, nodakenin is less well-known in the field. I investigated the anti-tumor effects of nodakenin in vivo and in vitro and showed that nodakenin exerts a powerful anti-cancer effect via the inhibition of cell viability and tumor volume and the enhancement of caspase-3 activity and LDH cytotoxicity. Furthermore, nodakenin activates apoptosis and the ER stress pathway with intracellular ROS and Ca^2+^ production. The accumulation of intracellular ROS and Ca^2+^ stimulates oxidative phosphorylation and induces apoptotic signaling via ER stress [49]. ER stress, caused by ROS and Ca^2+^ production from the ER lumen, mediates cell death via various signals, including cell cycle arrest, caspases, transcription factors, and the Bax/Bcl-2 family [50].

Radiotherapy has been used to treat breast cancer patients, and it is a powerful strategy to lower the risk of recurrence after surgery [51]. However, in breast cancer, radiation therapy often induces chemoresistance. Many reports suggest that combination therapy is a latent tumor therapeutic approach to overcome radioresistance for cancer patients [52]. Accumulating evidence indicates that EMT induces radioresistance through the loss of adhesion, decreased E-cadherin and increased vimentin, myosin, and N-cadherin [53]. Therefore, I investigated the synergistic effect of nodakenin and radiation in radioresistant breast cancer models, MCF-7R and MDAMB231R. Nodakenin with radiation overcame radioresistance by enhancing E-cadherin and blocking N-cadherin and vimentin expression.

Increasing reports suggest that an excessive ER stress response is a novel therapeutic strategy to overcome radioresistance [54]. In tumors, the ER stress inducer tunicamycin causes radiosensitization, inducing apoptotic cell death and suppressing EGFR phosphorylation on the ER and Golgi [55,56]. The combination treatment with tunicamycin and EGFR-TKI erlotinib induces cleaved caspase-3 and PARP via the activation of the pro-apoptotic ER stress marker CHOP. Then, the combination treatment mediates apoptotic cell death by increasing death receptor 5 (DR5) [57,58]. Our results showed that nodakenin treatment induced apoptotic cell death and the ER stress response via intracellular ROS and Ca^2+^ generation and the upregulation of p-PERK, GRP78, p-eIF2α, CHOP, and ATF4. Based on these findings, I suggest that nodakenin treatment may overcome radioresistance and induce programmed cell death through an excessive ER stress response in breast cancer.

## 5. Conclusions

In conclusion, I identified the anti-cancer effect of nodakenin in vivo and in vitro. Nodakenin induced apoptosis and upregulated ER stress-related proteins (the phosphorylation of PERK and eIF2α and the expression of GRP78, CHOP, and ATF4) via the upregulation of Nox4 and ROS and Ca^2+^ release in breast cancer cells. Moreover, I established radioresistant breast cancer models (MCF-7R and MDAMB231R) from MCF-7 and MDAMB231 cells and used a combined treatment with nodakenin and radiation to overcome radioresistance through the suppression of the EMT process, indicating the inhibition of E-cadherin and the enhancement of vimentin and N-cadherin.

## Data Availability

Not applicable.
